# Effects of Three Consecutive Days of Diurnal Intermittent Fasting on Cognitive Function, Psychomotor Vigilance, and Sleep Quality in Adolescent Male Soccer Players: A Quasi-Experimental Parallel-Group Study

**DOI:** 10.3390/nu18152475

**Published:** 2026-07-30

**Authors:** Hamza Baati, Sabra Ouni, Fida Ayachi, Feriel Amor, Mevlüt Yıldız, Halil İbrahim Ceylan, Raul Ioan Muntean, Omaima Chrif, MoezAlIslam E. Faris, Wissem Dhahbi

**Affiliations:** 1 Education, Motricité, Sport et Santé (EM2S), UR15JS01, High Institute of Sport and Physical Education, University of Sfax, Sfax 3000, Tunisiaouni.sabra89@gmail.com (S.O.);; 2 Research Unit (UR22JS01) “Sport Sciences, Health and Movement”, High Institute of Sport and Physical Education of Kef, University of Jendouba, El Kef 7100, Tunisiawissem.dhahbi@gmail.com (W.D.); 3 Interdisciplinary Laboratory in Neurosciences, Physiology and Psychology: Physical Activity, Health and Learning (LINP2), UFR STAPS (Faculty of Sport Sciences), Paris Nanterre University, 92000 Nanterre, France; ferielamor14000@gmail.com; 4 Coaching Sciences, Faculty of Sports Sciences, Mugla Sitki Kocman University, Mugla 48000, Türkiye; 5 Physical Education and Sports Teaching Department, Faculty of Sports Sciences, Atatürk University, Erzurum 25240, Türkiye; 6 Department of Physical Education and Sport, Faculty of Law and Social Sciences, University “1 Decembrie 1918” of Alba Iulia, 510009 Alba Iulia, Romania; 7 Department of Clinical Nutrition and Dietetics, Faculty of Allied Medical Sciences, Applied Science Private University, Amman 11931, Jordan; moezalislam@gmail.com; 8 Training Department, Police College, Qatar Police Academy, Doha 7157, Qatar

**Keywords:** adolescent, athletic performance, circadian rhythm, dehydration, neuropsychological tests, nutritional status, reaction time, sleep deprivation

## Abstract

**Aim:** While voluntary Islamic intermittent fasting (IF) of three consecutive days (mimicking practices such as Ayyam al-Bid) may be observed by Muslim athletes, its short-term effects on cognitive function and sleep quality in adolescent team-sport athletes remain unexamined. This study investigated the impact of three successive days of dawn-to-sunset IF on memory, attention, psychomotor vigilance, and subjective sleep quality in young male soccer players. **Methods:** A quasi-experimental, parallel-group design was employed. Thirty-five male soccer players (age: 15.6 ± 0.8 years; body mass: 55.07 ± 6.53 kg) were allocated to an IF group (*n* = 17) or a non-fasting control group (*n* = 18). The fasting protocol comprised approximately 14 consecutive hours of complete food and fluid abstinence per day (from 04:40 to 18:36, with daily variations of −3 min for the dawn and +2 min for the sunset, local time). Cognitive function was assessed using the CogniFit Cognitive Assessment Battery (CAB) and the Psychomotor Vigilance Test (PVT), and subjective well-being was quantified via the Hooper Index. Both assessment sessions were conducted between 14:00 and 17:00 to control for diurnal variation. **Results:** The Group × Time interaction for single-item subjective sleep quality reached nominal significance at the unadjusted threshold (Z = 2.18, *p* = 0.029, r = 0.37) but did not survive Bonferroni correction for the four Hooper Index comparisons (α = 0.0125); this result is therefore interpreted as hypothesis-generating only, reflecting a within-group deterioration in the control group (*p* < 0.05) alongside a non-significant marginal improvement in the fasting group. No statistically significant Group × Time interaction was detected for any memory subdomain (all *p* ≥ 0.419, r ≤ 0.14), attention component (all *p* > 0.176, r ≤ 0.23), or PVT parameter (all *p* > 0.192, η^2^*p*/r ≤ 0.22) under the present study conditions. Within-group improvements in memory and attention observed in both groups were consistent with test–retest learning effects. **Conclusions:** No statistically significant Group × Time interaction was detected for memory, attention-related executive functions, or psychomotor vigilance in young male soccer players under the present study conditions; the study was not designed as an equivalence or non-inferiority trial and cannot exclude effects smaller than the targeted medium magnitude, alongside a nominally divergent but statistically uncorrected sleep quality trajectory relative to non-fasting controls, which should be interpreted as hypothesis-generating pending replication. These findings provide preliminary, hypothesis-generating evidence that no statistically significant cognitive or psychomotor impairment was detected under the present study conditions; because the study was not designed as an equivalence or non-inferiority trial, compatibility between short-term voluntary IF and adolescent athletic training cannot be inferred from the absence of a significant interaction alone.

## 1. Introduction

Intermittent fasting (IF) encompasses a spectrum of dietary protocols characterized by structured alternation between periods of voluntary food and fluid abstinence and unrestricted dietary intake [1]. Practiced forms include time-restricted eating, alternate-day fasting, and periodic fasting [2,3]. Among Muslim populations, IF extends beyond Ramadan to year-round observance, most notably the voluntary three-day Ayyam al-Bid (the bright full-moon days) fast prescribed on lunar calendar days 13, 14, and 15 of each month [4]. As the global Muslim athlete population grows and competitive training schedules increasingly overlap with religious observances by many Muslim athletes, quantifying the physiological and neuropsychological consequences of IF on athletic populations has become an urgent scientific priority [5,6].

Soccer is a cognitively demanding sport in which performance depends substantially on intact executive function, sustained attention, rapid decision-making, visuospatial working memory, and psychomotor speed [7,8]. Tactical positioning, anticipatory scanning, and response selection under time pressure require athletes to maintain high cognitive throughput across match durations that can exceed ninety minutes [9]. Any dietary or chronobiological perturbation that compromises attentional control, reaction speed, or memory consolidation therefore carries direct adverse consequences for both performance and injury risk. These concerns are amplified in adolescent athletes, whose prefrontal cortical networks supporting executive function remain developmentally incomplete and may exhibit differential susceptibility to metabolic and sleep-related stressors relative to adults [10].

The cognitive consequences of Ramadan IF (RIF) in athletic populations have been investigated across several domains, yielding contradictory and context-dependent results. Boujelbane et al. [11] reported that physically active individuals undergoing four weeks of RIF demonstrated significant improvements in executive function (*p* = 0.035), attention (*p* = 0.005), inhibition (*p* = 0.02), and associative and recognition memory (*p* < 0.05). In contrast, sedentary participants showed cognitive decline over the same period. By contrast, Bougrine et al. [12] observed a progressive and significant deterioration in simple reaction time, choice reaction time, vigilance, and mental rotation in adolescent female handball players across four weeks of Ramadan, with the most pronounced impairments recorded during the fourth week. A central mechanism linking IF to cognitive disruption is alteration of sleep architecture. Diurnal fasting shifts the timing of caloric intake toward nocturnal hours, compressing or delaying sleep windows and reducing the proportion of rapid eye movement (REM) sleep [13]. Trabelsi et al. [5] confirmed in a systematic review and meta-analysis that RIF is reliably associated with reduced nocturnal sleep duration and impaired subjective sleep quality among athletes who continue to train during the fasting month. Sleep restriction, in turn, impairs vigilance, executive function, and working memory through prefrontal hypoactivation and dysregulation of the adenosine-driven sleep homeostatic system [6,14]. The variability in reported cognitive outcomes across studies is attributable to methodological heterogeneity including fasting duration, sample sex and training status, time-of-day of testing, last meal timing (Suhoor), and the magnitude of lifestyle disruption adopted by individual athletes and coaching staff [12].

Critically, the existing literature is dominated by studies of RIF, a protocol of 29 to 30 consecutive days with substantial cumulative sleep debt and behavioral disruption. The short-term Islamic IF model, specifically the three-day Ayyam al-Bid observance practiced outside Ramadan with structurally controlled daily routines, has received minimal scientific attention. Cherif et al. [4] examined this protocol in male athletes. They reported no significant impairment of working memory, spatial planning, or executive function, alongside a reduction in repeated-sprint performance attributable to vertical stiffness changes. However, that investigation did not comprehensively evaluate memory subdomains, omitted computerized assessment of psychomotor vigilance, and enrolled adult rather than adolescent participants. Whether three consecutive dawn-to-sunset fasting days produce differential effects on memory, attention-related executive functions, and psychomotor vigilance specifically in young soccer players, a population for whom both cognitive and physical demands intersect with developmental vulnerability, remains unresolved.

This study therefore aimed to determine the association between three consecutive days of diurnal IF, approximating the Ayyam al-Bid protocol, and memory, attention, psychomotor vigilance, and subjective sleep quality in male adolescent soccer players, using a quasi-experimental parallel-group design with standardized pre-fasting nutrition. Given the short duration and controlled pre-dawn macronutrient intake, we anticipated that any fasting-related change in cognitive and psychomotor outcomes over three days would be modest and potentially undetectable at the achieved sample size. At the same time, sleep quality trajectories were expected to diverge between fasting and non-fasting conditions through circadian scheduling effects. This study was designed to detect group differences, not to establish equivalence between fasting and non-fasting conditions.

## 2. Methods

### 2.1. Participants

A priori power analysis was conducted in G*Power version 3.1.9.4 [15] and determined a minimum required sample of 34 participants to achieve adequate statistical power. The analysis specified a medium effect size (f = 0.25), a two-tailed alpha of 0.05, a desired power of 0.80, and a repeated-measures mixed design with two time points, assuming a within-measure correlation of r = 0.50 and a non-sphericity correction epsilon of 1.00. The adopted effect size was informed by effect magnitudes consistently reported in structurally comparable investigations of short-term IF on cognitive and psychomotor outcomes in trained athletes, where medium-range effects were observed for attention, memory, and vigilance [4,16,17]. The final enrolled sample of 35 participants therefore met and marginally exceeded the computed minimum threshold. This calculation targeted a medium effect size (f = 0.25); consequently, the achieved sample provides adequate sensitivity only for effects of this magnitude or larger, and smaller interaction effects in the r ≈ 0.20–0.23 range, such as those observed for updating and focused attention (Section 3.4), likely fall below the detectable threshold at this sample size.

Thirty-five male soccer players (age: 15.6 ± 0.8 years; body mass: 55.07 ± 6.53 kg; height: 1.70 ± 0.06 m; training experience: 3.89 ± 2.89 years) from a single civilian club (OCK) volunteered for this study. Inclusion criteria were: (i) male sex; (ii) age between 14 and 17 years; (iii) an active competitive training status of approximately eight hours per week, inclusive of school PE and match play; (iv) absence of chronic medical, neurological, or psychiatric conditions; (v) normal or corrected-to-normal vision; and (vi) Muslim faith with prior experience or willingness to adhere to the fasting protocol. Exclusion criteria comprised current use of pharmacological agents known to influence cognitive performance or sleep architecture, a clinical diagnosis of a sleep disorder, and a body mass index outside the age-appropriate healthy reference range for male adolescents [18]. Players occupied multiple positional roles, including defenders, midfielders, strikers, and goalkeepers.

The study protocol was formally reviewed and approved by the Ethics Committee of the Hédi Chaker University Hospital of Sfax, Tunisia (Reference No. CE 10/2025; approved 17 February 2025). Before the study commenced and under the supervision of the team coach, all participants received a comprehensive explanation of the experimental procedures, potential risks, and the voluntary nature of the research. Informed consent and assent were obtained from both parents and the young athletes, with the explicit right to withdraw at any point without penalty. All methodological procedures were conducted in strict adherence to the ethical principles for medical research involving human subjects, as stipulated in the Declaration of Helsinki [19]. Participants received no financial remuneration or incentives for their involvement in the study.

### 2.2. Study Design

This investigation employed a quasi-experimental, parallel-group design with two measurement sessions: a baseline assessment under habitual feeding conditions and a post-intervention assessment conducted after three consecutive days of either intermittent fasting (IF) or normal dietary intake. Participants were allocated to the IF group (*n* = 17) or the non-fasting control group (*n* = 18) according to their voluntary agreement to complete the three consecutive days of the experimental fasting protocol. This naturalistic allocation strategy preserved ecological validity and replicated real-world fasting conditions in young Muslim athletes [4]. However, allocation by declared fasting intention rather than randomization precludes control for unmeasured confounders (e.g., motivation, habitual sleep behavior, dietary discipline) that may correlate with the decision to fast, limiting causal attribution of the observed group differences. To reduce assessment bias, outcome assessors administered all neuropsychological and psychomotor tests without prior knowledge of group assignment, constituting a single-masked outcome evaluation. To accommodate the athletes’ academic and training schedules, as well as their arrival order, testing was conducted individually or in small staggered groups, ensuring that each participant completed the full battery of paper-based questionnaires and computerized tests (CogniFit and PVT) under identical, standardized environmental conditions. A familiarization session was administered before baseline testing to attenuate learning and practice effects on cognitive assessments. All testing sessions were conducted between 14:00 and 17:00 local time to control for diurnal variation in cognitive performance and psychomotor vigilance, consistent with chronobiological evidence that time-of-day exerts a robust effect on executive function and reaction speed [9,20,21]. To prevent assessment bias, all cognitive and psychomotor tests were administered while participants were in a rested state.

### 2.3. Procedures

The experimental protocol unfolded across three distinct phases: a familiarization session, a baseline assessment, and a post-intervention assessment. Conducted before formal testing, this initial phase ensured that participants gained adequate exposure to the cognitive and psychomotor tests, thereby mitigating potential learning or practice effects during subsequent assessments. The baseline assessment was then administered under habitual feeding conditions and served as the reference point against which post-intervention change was evaluated. Following baseline, participants entered the three-day intervention period, during which the IF group observed the prescribed fasting protocol and the control group maintained habitual dietary behavior. On the final day of the intervention, the post-intervention assessment session was conducted. To manage testing fatigue and safeguard data integrity, the assessment flow transitioned strategically from paper-based self-reports to computerized neurocognitive testing. Participants completed the subjective Hooper Index (HI) before engaging in the computerized cognitive and psychomotor batteries (CogniFit and PVT), thereby minimizing the confounding effects of progressive exhaustion on self-reported metrics. All sessions were held between 14:00 and 17:00 local time in a quiet, temperature-controlled room (22 ± 2 °C) under standardized ambient lighting. To ensure data validity, all neurocognitive assessments were systematically conducted while participants were fully rested.

#### 2.3.1. Dietary Protocol and Hydration Monitoring

The IF group followed a diurnal fasting protocol of approximately 14 consecutive hours per day (from 04:40 to 18:36, with daily variations of −3 min for the dawn and +2 min for the sunset, local time) across three days, abstaining completely from food and fluid during the fasting window. To reduce inter-individual variability in pre-fast nutritional status, a standardized Suhoor meal was prescribed and physically provided to all IF-group participants on each fasting day. The meal was adapted from the macronutrient framework of Aziz et al. [22], which recommends approximately 3502 ± 404 kJ (837 ± 96.5 kcal) with 2.0 g/kg body mass of complex carbohydrates and 0.5 g/kg of protein. Meals were prepared and distributed under researcher supervision before the onset of fasting. Consumption was monitored through the participants’ compliance; however, individual food intake was not weighed or biochemically verified, and the reported macronutrient values represent prescribed targets rather than confirmed achieved intakes. Compliance was assessed through self-adherence and researcher inquiry, and no participant reported deliberate non-compliance; nonetheless, inter-individual variability in actual intake cannot be excluded and constitutes a limitation on the dietary control achieved in this study. The control group maintained their habitual dietary patterns, hydration behaviors, and daily routines throughout the study duration.

#### 2.3.2. Subjective Well-Being: Hooper Index

Subjective well-being was assessed at each session using the HI [23], a validated self-report instrument comprising four items: sleep quality, perceived stress, fatigue, and delayed-onset muscle soreness (DOMS). Each item was rated on a 7-point Likert scale anchored at 1 (very low or very good) and 7 (very high or very bad), with a higher composite score indicating greater subjective burden. Participants completed the HI immediately before commencing the cognitive test battery. The instrument demonstrates adequate responsiveness to short-term fluctuations in training and dietary load in competitive athletes [23]. Sleep quality was assessed via a single Likert item within the HI rather than a validated multidimensional instrument; sleep is increasingly recognized as a multidimensional construct requiring convergent subjective and objective assessment [24]. Accordingly, HI sleep scores in the present study index short-term subjective perception rather than sleep duration, efficiency, or architecture.

#### 2.3.3. Neuropsychological Assessment: CogniFit Cognitive Assessment Battery

Cognitive function was quantified using the CogniFit Cognitive Assessment Battery (CAB; CogniFit Inc., San Francisco, CA, USA), a validated computerized neuropsychological platform standardized for individuals aged seven years and older [25]. Scores are expressed on a 0–800-point continuum, with higher values reflecting superior cognitive performance. The CAB evaluates memory subdomains, including phonological short-term memory, working memory, contextual memory, visual short-term memory, naming, and non-verbal memory, alongside attention subdomains, including divided attention, focused attention, inhibitory control, and updating, yielding aggregate memory and attention indices. Concurrent validity has been established against several gold-standard neuropsychological instruments, including the Cambridge Neuropsychological Test Automated Battery (CANTAB), the Wisconsin Card Sorting Test, the Stroop Color-Word Test, Raven’s Standard Progressive Matrices, and the Continuous Performance Test [25,26,27]. All assessments were conducted on laptop computers using the standardized CogniFit platform in a quiet, temperature-controlled room (22 ± 2 °C) under standardized ambient light conditions to minimize environmental variability. Automated score exports were audited for out-of-range or zero-variance values before analysis. The zero-variance baseline value encountered in the fasting group’s divided attention subdomain was verified against the source data export and handled using non-parametric statistical analyses; the explanation offered in Section 3.4 is based on direct inspection of the raw data and the CogniFit platform’s automated output, as no independent or publicly available technical documentation of this scoring rule was identified.

#### 2.3.4. Psychomotor Vigilance: PVT

Alertness and psychomotor vigilance were assessed using a validated two-minute, computerized version of the Psychomotor Vigilance Test (PVT) [28]. Participants fixated on a rectangular stimulus box displayed centrally on screen. They were instructed to press a designated response key as rapidly as possible upon presentation of a red numerical counter, which appeared at unpredictable inter-stimulus intervals. Primary outcome variables were test duration (seconds), number of false starts (response latency < 100 ms), mean response time (ms), and total number of valid attempts. False-start responses were excluded from mean response time calculations. The PVT demonstrates well-established sensitivity to sleep restriction, circadian disruption, and fatigue-related vigilance decrements in athletic and non-athletic populations alike [14,21].

### 2.4. Statistical Analysis

All analyses were conducted in Statistica 12 (StatSoft Inc., Tulsa, OK, USA), with statistical significance set at *p* < 0.05. Data are expressed as means ± standard deviations. Departure from normality was evaluated for each variable using the Shapiro–Wilk test before inferential analysis. For variables satisfying normality assumptions, a two-way mixed-design analysis of variance (ANOVA) was applied, with Group (fasters vs. non-fasters) as the between-subjects factor and Time (pre vs. post) as the repeated within-subjects factor. Partial eta squared (η^2^*p*) quantifies effect magnitude for all parametric omnibus tests, with values of 0.01, 0.06, and 0.14 interpreted as small, medium, and large, respectively [29]. For significant Group × Time interactions, pairwise comparisons were performed with Bonferroni correction to control the family-wise error rate. For variables violating normality, within-group pre-to-post differences were assessed using the Wilcoxon signed-rank test, and between-group comparisons at each time point and for delta scores were assessed using the Mann–Whitney U test. Effect sizes for non-parametric comparisons are reported as rank-biserial correlation (r), with thresholds of 0.10, 0.30, and 0.50 representing small, medium, and large effects [30]. Delta scores (delta = post minus pre) and percentage change (delta% = [delta/pre] × 100) were computed per participant to quantify individual-level change. Sleep quality, memory, attention, and psychomotor vigilance constituted four a priori, conceptually distinct primary constructs; no correction for multiplicity was applied across constructs, as the corresponding contrasts were directed and specified independently of the data rather than derived from an undirected outcome screen [31]. This strategy does not remove the elevated family-wise error risk inherent in evaluating multiple subdomain-level tests jointly; isolated significant findings are therefore interpreted as hypothesis-generating (see Section 4). Benjamini–Hochberg false discovery rate control was considered as an alternative to family-wise correction but was not applied, because the four outcome domains were treated as separate pre-specified constructs rather than as a single pool of exploratory contrasts competing for discovery [31]. Listwise deletion was applied to any incomplete cases, given the low anticipated attrition rate.

## 3. Results

### 3.1. Participant Flow and Baseline Characteristics

All 35 participants completed both assessment sessions with complete data (IF group: *n* = 17; control group: *n* = 18). Baseline comparisons revealed no statistically significant between-group differences across all HI subscales, CogniFit memory and attention subdomains, and PVT metrics (all *p* > 0.05). Numerical between-group differences, including a higher phonological short-term memory score in the IF group relative to controls at Session 1 (400.0 ± 175.2 vs. 288.7 ± 178.4), did not reach statistical significance and should not be interpreted as pre-intervention equivalence; they are acknowledged as a potential limitation on the interpretive validity of subsequent Group × Time interaction analyses.

### 3.2. Subjective Well-Being: Hooper Index

The Group × Time interaction for sleep quality reached the unadjusted significance threshold (Z = 2.18, *p* = 0.029, r = 0.37; Table 1) but did not survive Bonferroni correction for the four Hooper Index comparisons (α = 0.0125) and is therefore reported as hypothesis-generating only. Non-fasters exhibited a significant within-group deterioration in sleep scores across the intervention period (*p* < 0.05, Wilcoxon signed-rank test). In contrast, the IF group showed a non-significant marginal improvement (*p* > 0.05). The effect size (r = 0.37) represents a small-to-medium magnitude, reflecting divergent trajectories rather than a clinically distinct endpoint, as absolute scores at Session 2 did not differ significantly between groups (*p* > 0.05). No significant Group × Time interaction was detected for perceived stress, fatigue, or DOMS (all *p* > 0.704, all r < 0.06; Table 1). Both groups exhibited parallel significant within-group increases in fatigue and DOMS (all *p* < 0.05), attributable to cumulative training load rather than dietary condition.

### 3.3. Memory Function

No significant Group × Time interaction was identified for any CogniFit memory subdomain (all *p* > 0.05; Table 2). Interaction effect sizes were uniformly negligible to small (r range: 0.00–0.14), consistent with the absence of a detectable fasting-specific effect at the achieved sample size; however, effects smaller than the targeted medium magnitude (f = 0.25) cannot be excluded given the study’s power specification. Both groups demonstrated parallel significant within-group improvements across contextual memory, naming, visual short-term memory, and the composite memory score (all *p* < 0.05, Wilcoxon signed-rank test; Table 2), a pattern most parsimoniously attributed to test–retest learning effects given its consistency across conditions and the absence of any significant interaction. The higher phonological short-term memory score observed in fasters at Session 2 reflected a numerical baseline differential present at Session 1 (fasters: 400.0 ± 175.2 vs. non-fasters: 288.7 ± 178.4; Table 2) that did not reach statistical significance; it does not indicate a fasting-induced change, and the Group × Time interaction for this subdomain was negligible (*p* = 0.982, r = 0.00).

### 3.4. Attention Function

No significant Group × Time interaction was detected for any attention subdomain (all *p* > 0.05; Table 3). Effect sizes for interaction terms ranged from r = 0.03 (divided attention, inhibition) to r = 0.23 (updating); the latter, while statistically non-significant (*p* = 0.176), represents a medium-range magnitude and is reported here as an exploratory, hypothesis-generating signal warranting replication in adequately powered samples, rather than as evidence of a true fasting effect. For updating and inhibition, significant within-group improvements were observed across both groups (all *p* < 0.05; Table 3), a pattern that diverged only for divided attention, where non-fasters exhibited a significant decline (*p* < 0.05) while fasters improved (*p* < 0.05), rendering the overall performance gains consistent with a practice effect operating independent of dietary condition. The divergent within-group patterns for divided attention cannot be attributed to the fasting manipulation: Group × Time interaction was negligible (r = 0.03, *p* = 0.869), and the zero-variance baseline in the IF group (all *n* = 17 scored 8/800, SD = 0) renders within-group change in that group uninterpretable, because any post-intervention score necessarily exceeds the floor irrespective of dietary condition. Differences in within-group significance patterns should not be construed as evidence of between-group differences [31]. A directional divergence for focused attention, where non-fasters improved significantly (*p* < 0.05) and fasters declined non-significantly, produced a medium-range but non-significant interaction (Z = 1.27, *p* = 0.204, r = 0.21). The significantly lower composite attention score in fasters at Session 2 (*p* < 0.05) partially reflected a pre-existing performance differential visible at baseline (Table 3), and the non-significant interaction (*p* = 0.338, r = 0.16) precludes attribution of this cross-sectional gap to the fasting manipulation. The baseline standard deviation of zero (SD = 0) for the divided attention variable within the fasters group (*n* = 17) was verified against the raw data export; all 17 participants scored 8/800 at Session 1. This uniform score was treated as a psychometric floor effect attributable to the CogniFit CAB platform’s automated output under high cognitive overload conditions; however, no independent or publicly available technical documentation of this specific scoring rule was identified. The explanation is based solely on direct inspection of the platform output, and this limitation should be considered when interpreting within-group change for this subdomain. Non-parametric analyses were applied to account for this distributional anomaly.

### 3.5. Psychomotor Vigilance

No significant Group × Time interaction was identified for any PVT parameter (all *p* > 0.05; Table 4). Interaction effect sizes were small to trivial for test duration (η^2^*p* = 0.01), false starts (r = 0.02), and mean reaction time (r = 0.05). Based on within-group non-parametric analyses, false starts increased significantly within both groups from Session 1 to Session 2 (both *p* < 0.05; Table 4), indicating a generalized shift toward impulsive responding across conditions, consistent with cumulative fatigue and sleep disruption irrespective of dietary manipulation. Non-fasters achieved a significant within-group reduction in mean reaction time (*p* < 0.05). In contrast, the comparable magnitude of improvement in fasters did not reach significance, likely due to substantially greater inter-individual variability in the delta scores of that group (Δ SD = 94.0 ms vs. 44.9 ms; Table 4). The divergent pattern in total attempts, where non-fasters increased, and fasters remained stable, yielded the largest PVT interaction effect size (r = 0.22, *p* = 0.192), suggesting a possible accuracy-prioritization strategy in the fasting condition; however, the absence of a significant interaction precludes a definitive interpretation.

## 4. Discussion

The primary aim of this study was to examine the association between three consecutive days of diurnal IF and memory, attention-related executive functions, psychomotor vigilance, and subjective sleep quality in young male soccer players. The central finding is that a short-term, dawn-to-sunset IF protocol of approximately 14 h per day produced no significant differential effect on any measured cognitive or psychomotor parameter, while generating a nominally significant, unadjusted Group × Time divergence in single-item sleep quality scores (Z = 2.18, *p* = 0.029, r = 0.37) that did not survive Bonferroni correction (alpha = 0.0125) and is therefore interpreted as hypothesis-generating only. These findings address an underexamined question in the sports nutrition literature: whether voluntary short-term Islamic IF, distinct in duration and context from the widely studied RIF model, is associated with detectable cognitive impairment in adolescent athletes under the present testing conditions.

The nominally significant but uncorrected Group x Time interaction for sleep quality (*p* = 0.029, not retained after Bonferroni correction) reflected a selective deterioration in the non-fasting control group, whose Hooper sleep scores worsened across the intervention period (Delta = +0.44, 95% CI [−0.08, +0.96]), while the IF group maintained stable or marginally improved scores (Delta = −0.29, 95% CI [−0.76, +0.18]); this pattern is interpreted as hypothesis-generating rather than confirmatory. If replicated in adequately powered randomized trials, this pattern would be counterintuitive relative to the established Ramadan IF literature, which consistently documents sleep disruption through delayed bedtimes, reduced total sleep time, and diminished REM proportion [5,13]. One plausible explanation is that the standardized Suhoor schedule imposed a temporally stable pre-dawn anchor in the IF group, supporting circadian entrainment and reducing inter-individual variability in sleep timing [32]. In contrast, the control group’s unrestricted schedules may have permitted greater nocturnal behavioral variability, manifesting as a subjective sleep decrement by day three. The effect size was small-to-medium (r = 0.37), and Session 2 absolute scores did not differ significantly between groups, indicating that neither group experienced clinically severe sleep impairment over the three-day window; the divergence in trajectories therefore remains a directional, uncorrected signal requiring prospective confirmation. A further consideration is that some participants may have had prior experience with diurnal intermittent fasting (such as during Ramadan), which could have potentially attenuated the psychobiological disruption that accompanies novel circadian realignment. This interpretation is consistent with evidence that experienced fasters exhibit attenuated sleep disturbance compared to those newly observing IF protocols [10].

No statistically significant Group × Time interaction was detected across any memory subdomain under the present conditions, with effect sizes ranging from r = 0.00 to r = 0.14. This is consistent with Cherif et al. [4], who similarly detected no impairment in working memory or executive planning after an identical three-day IF protocol; however, neither study was designed as an equivalence trial, and smaller effects cannot be excluded. The parallel within-group improvements observed for contextual memory, visual short-term memory, naming, and the composite memory score in both groups most likely reflect standard practice effects and progressive familiarity with the testing procedures since the baseline assessment, rather than genuine fasting-induced enhancement. As an exploratory, hypothesis-generating explanation not directly tested in the present data, the preservation of memory function during short-term IF may be related to a metabolic transition toward ketone body utilization. After approximately three days of caloric restriction, the brain reportedly derives roughly 30% of its energy from ketone bodies, primarily beta-hydroxybutyrate, which may stimulate brain-derived neurotrophic factor (BDNF) expression [33], a pathway implicated in synaptic plasticity and hippocampal neurogenesis in prior mechanistic work. Whether this pathway operated in the present cohort could not be verified, as neither ketone body nor BDNF concentrations were measured; this mechanism should therefore be regarded as speculative pending direct biochemical assessment.

No statistically significant Group × Time interaction was detected for any attention subdomain under the present study conditions. The largest interaction effect emerged for updating (r = 0.23, *p* = 0.176), which, while non-significant, reflects a medium-range magnitude that warrants replication in adequately powered designs. The directional divergence in focused attention, where non-fasters improved significantly (Δ = +70.9, 95% CI [−17.99, +159.79]) and fasters showed a non-significant decline (Δ = −29.4, 95% CI [−133.62, +74.82]), may be consistent with progressive depletion of circulating glucose in the late afternoon, given the brain’s reliance on peripheral glucose input during the final hours of a diurnal fast [20]; however, this glucose-based explanation is speculative, as no blood glucose measurements were obtained. However, the absence of a significant interaction (*p* = 0.204) and the relatively modest effect size (r = 0.21) suggest that this substrate-related vulnerability did not translate into a measurable performance decrement under the present conditions. Bougrine et al. [10] reported progressive and significant deterioration in cognitive and psychomotor parameters across the four weeks of Ramadan in female adolescent athletes, with the most pronounced decrements during the fourth week. The contrast with the present null finding is instructive: it underscores the critical role of cumulative duration in determining whether IF-induced circadian and metabolic perturbations reach the threshold of cognitive impairment. Over three days, compensatory metabolic and circadian adaptation appears sufficient to preserve attentional function. The within-group gains observed in both groups for inhibition and updating are attributed to test–retest learning; because these practice-related gains occurred irrespective of dietary condition, they may also have partially masked a smaller, genuine fasting-related decrement, reducing the sensitivity of the present design to detect subtle Group × Time effects in these specific subdomains. Conversely, the divergent within-group patterns for divided attention (IF group improvement; control group decline) are uninterpretable as evidence of a fasting-specific effect given the negligible Group × Time interaction (r = 0.03), the non-significant between-group comparison, and the zero-variance floor score in the IF group at baseline (all fasters scored 8/800, SD = 0), which precludes meaningful within-group change estimation for that group.

The absence of a statistically significant Group × Time interaction for all PVT parameters indicates that no differential effect of three days of IF on alertness or psychomotor speed was detectable under the present study conditions; this does not constitute evidence of equivalence between fasting and non-fasting conditions. Both groups showed a significant within-group increase in false starts (*p* < 0.05), a pattern consistent with the progressive accumulation of training fatigue and mild vigilance degradation across repeated afternoon testing sessions, irrespective of dietary condition [14,21]. The comparable reduction in mean reaction time across both groups, significant only in non-fasters due to lower inter-individual variability in the delta scores (SD: 44.9 ms vs. 94.0 ms for fasters), may reflect genuine practice-related improvements in stimulus anticipation. Tian et al. [17] observed time-of-day effects on psychomotor function and verbal memory during RIF in male athletes, with poorer performance at 16:00 relative to 09:00 (d = −0.4 to −1.3). The present study tested all participants between 14:00 and 17:00, corresponding to the most metabolically vulnerable window of a diurnal fast. The null interaction finding under these conditions is consistent with the absence of a detectable fasting-related effect on psychomotor vigilance under the present testing conditions. However, the study was not designed to confirm equivalence between groups.

Several methodological constraints qualify the present conclusions. The quasi-experimental allocation strategy, though ecologically valid, did not permit randomization, introducing potential selection bias. Athletes who elect to fast may differ systematically from non-fasters in motivation, self-discipline, or baseline sleep habits, any of which could independently influence cognitive and psychomotor outcomes; consequently, the reported associations should not be interpreted as causal effects of fasting per se. The sample comprised exclusively young male athletes, limiting generalizability to other athletic cohorts, non-Muslim populations, or those with different training backgrounds. Sleep was assessed subjectively via the HI; objective quantification through actigraphy or polysomnography would provide a more sensitive and valid estimate of sleep architecture changes. Even validated multi-item subjective instruments show limited convergent validity against actigraphy in athletic populations [34], underscoring the need for cautious interpretation of the single-item HI sleep score reported here. A single-item measure was selected over a multi-item sleep diary as it inherently minimizes respondent burden across the fixed three-day testing window and maintains consistency with the brief athlete-monitoring format of the HI [23]; this choice, however, precluded concurrent assessment of sleep duration, which may interact with perceived sleep quality. Future studies should consider randomized allocation where ethically and religiously feasible, or, where naturalistic allocation is required, statistical adjustment (e.g., propensity-score matching on baseline motivation and training history) to mitigate confounding by indication. Neither blood glucose nor urine osmolality was measured in this study, precluding direct examination of glucose availability or urine osmolality-derived hydration status as potential confounders of the cognitive and psychomotor outcomes. The zero-variance baseline in the fasters’ divided attention task (SD = 0) was verified against the raw data export; based on inspection of the platform output, this uniform score is interpreted as a potential psychometric floor effect under high cognitive overload conditions, though no independent or publicly available technical documentation of this specific CogniFit CAB scoring rule was identified. This explanation therefore cannot be independently verified. Additionally, the CogniFit CAB and PVT, although validated against established neuropsychological batteries [25,26,27], have not been specifically validated for sensitivity to the short-term (three-day), naturalistic dietary perturbation examined here; the possibility that these instruments lack sufficient sensitivity to detect subtle IF-related change cannot be excluded and should be addressed with fasting-specific sensitivity data in future work. Finally, the absence of a multiplicity correction across seventeen concurrent outcome tests increases the risk that the isolated significant sleep-quality finding reflects a Type I error rather than a genuine effect. Independently, the study was not powered to detect the interaction effect sizes observed for updating (r = 0.23) or focused attention (r = 0.21), raising the possibility that these non-significant results reflect Type II error rather than a true absence of effect.

### Practical Recommendations

The following considerations are drawn from the external literature and were not directly evaluated in the present study; they should not be interpreted as conclusions derived from the present data. Coaches and practitioners working with young Muslim athletes who observe voluntary short-term IF outside Ramadan may find no evidence, from the present quasi-experimental data, to anticipate statistically significant cognitive or psychomotor decrements across a three-day fasting block; this observation does not establish that fasting has no such effect and cannot be interpreted as an equivalence finding. Structured sleep hygiene protocols should be applied universally during training periods, as the present data indicate that non-fasting athletes are not exempt from training-period sleep deterioration; however, the basis for this observation is an unadjusted, hypothesis-generating result. Based on the broader literature on pre-dawn nutrition in Muslim athletes [10,12], Suhoor meals incorporating complex carbohydrates at approximately 2.0 g/kg body mass have been proposed as a strategy to sustain hepatic glycogen availability during prolonged diurnal fasting; this recommendation is derived from prior nutritional intervention studies and was not directly examined here. Evidence from chronobiology suggests that high-intensity cognitive or tactical training sessions may be better tolerated before mid-afternoon during fasting periods, owing to progressive glucose availability constraints in the late fasting window [21]; this scheduling consideration is external to the present findings. Objective sleep monitoring through wearable actigraphy has been recommended as the standard for season-long assessment of sleep duration and timing in athletic populations [6]; its use during fasting blocks would provide more precise evidence than the single-item subjective measure employed here.

## 5. Conclusions

Three consecutive days of dawn-to-sunset IF, modeled after the Islamic Ayyam al-Bid practice, were not associated with statistically significant Group × Time interactions for memory, attention-related executive functions, or psychomotor vigilance in young male soccer players tested in the late afternoon. A nominally significant divergence in single-item subjective sleep ratings was observed at the unadjusted threshold (Z = 2.18, *p* = 0.029, r = 0.37) but did not survive Bonferroni correction (α = 0.0125); this hypothesis-generating pattern, in which non-fasting controls reported worsening perceived sleep while fasters maintained stable scores, is restricted to short-term subjective perception and does not extend to sleep duration, efficiency, or architecture. These findings provide preliminary, hypothesis-generating evidence that no statistically significant Group x Time differences in cognitive or psychomotor outcomes were detected under the present study conditions; because the study was not designed as an equivalence or non-inferiority trial, smaller effects cannot be excluded and compatibility between short-term voluntary IF and adolescent athletic training cannot be inferred from the absence of a significant interaction alone. Future research should adopt randomized, equivalence or non-inferiority designs with a priori-defined margins to confirm whether short-term IF is cognitively neutral rather than merely undifferentiated from control at the present sample size. Incorporating objective sleep assessment, continuous glucose monitoring, and longer fasting durations would further clarify the threshold at which this apparent stability gives way to meaningful performance impairment.

## Figures and Tables

**Table 1:** Descriptive statistics and Group × Time interaction results for Hooper Index well-being parameters.

Parameter	Group	Session 1 Mean ± SD	Session 2 Mean ± SD	Δ (Post − Pre) Mean ± SD	95% CI for Δ	Test Statistic (Z or F)	Exact *p*	Effect Size (r or η^2^*p*)
Sleep quality	Non-fasters	3.11 ± 1.13	3.56 ± 0.98 ^a^	+0.44 ± 1.04	[−0.08, +0.96]	2.18	0.029	0.37
	Fasters	3.82 ± 1.24	3.53 ± 0.94	−0.29 ± 0.92 ^b^	[−0.76, +0.18]			
Perceived stress	Non-fasters	2.94 ± 0.94	2.89 ± 0.58	−0.06 ± 0.87	[−0.49, +0.37]	0.38	0.704	0.06
	Fasters	2.53 ± 0.94	2.59 ± 0.94	+0.06 ± 0.75	[−0.33, +0.45]			
Fatigue	Non-fasters	2.50 ± 0.79	3.17 ± 0.92	+0.67 ± 0.59	[+0.38, +0.96]	0.03	0.974	0.01
	Fasters	2.59 ± 1.28	3.29 ± 0.99	+0.71 ± 0.92	[+0.24, +1.18]			
DOMS	Non-fasters	2.56 ± 1.04	3.06 ± 0.64	+0.50 ± 0.79	[+0.11, +0.89]	0.02	0.987	0
	Fasters	2.53 ± 1.37	3.06 ± 1.20	+0.53 ± 0.87	[+0.08, +0.98]			

Notes. Values are means ± SD (7-point Likert scale; 1 = very low/very good, 7 = very high/very bad). *n*: non-fasters = 18, fasters = 17. All analyses were conducted on delta (Δ) scores using the Mann–Whitney U test; within-group pre-to-post changes were assessed by the Wilcoxon signed-rank test. Effect size r = rank-biserial correlation (small: 0.10, medium: 0.30, large: 0.50).^a^ Session 2 was significantly different from Session 1 within group (*p* < 0.05). ^b^ Δ score significantly different from non-fasters (*p* < 0.05). The sleep-quality Group × Time interaction (*p* = 0.029) did not survive Bonferroni correction (α = 0.0125) and is treated as a hypothesis-generating, unadjusted result.

**Table 2:** Descriptive statistics and Group × Time interaction results for CogniFit memory subdomains (scores 0–800).

Parameter	Group	Session 1 Mean ± SD	Session 2 Mean ± SD	Δ (Post − Pre) Mean ± SD	95% CI for Δ	Test Statistic (Z or F)	Exact *p*	Effect Size (r or η^2^*p*)
Phonological STM *	Non-fasters	288.7 ± 178.4	299.1 ± 163.8	+10.3 ± 276.5	[−127.86, +148.46]	0	0.982	0
	Fasters	400.0 ± 175.2	408.5 ± 153.9	+8.5 ± 164.9 ^b^	[−76.29, +93.29]			
Working memory *	Non-fasters	424.2 ± 117.1	479.1 ± 81.0	+54.9 ± 134.4	[−12.26, +122.06]	0.09	0.762	0
	Fasters	465.2 ± 112.1	504.7 ± 105.2	+39.5 ± 162.5	[−44.05, +123.05]			
Contextual memory	Non-fasters	498.4 ± 57.2	505.1 ± 30.5 ^a^	+6.6 ± 58.8	[−22.78, +35.98]	0.3	0.766	0.05
	Fasters	455.2 ± 87.9	460.9 ± 64.4 ^a^	+5.7 ± 111.6	[−51.68, +63.08]			
Naming	Non-fasters	616.7 ± 37.1	624.0 ± 17.7 ^a^	+7.3 ± 41.5	[−13.44, +28.04]	0.4	0.692	0.07
	Fasters	580.6 ± 67.1	591.0 ± 54.6 ^a^	+10.4 ± 89.0	[−35.36, +56.16]			
Composite Short-term memory	Non-fasters	364.7 ± 194.5	475.1 ± 169.5	+110.4 ± 241.8	[−10.42, +231.22]	0.07	0.79	0
	Fasters	462.9 ± 224.0	552.6 ± 162.9	+89.7 ± 214.4	[−20.54, +199.94]			
Visual STM	Non-fasters	407.8 ± 189.5	555.8 ± 150.3 ^a^	+148.0 ± 217.4	[+39.37, +256.63]	0.28	0.779	0.05
	Fasters	476.1 ± 247.4	588.1 ± 175.5 ^a^	+112.0 ± 270.9	[−27.29, +251.29]			
Non-verbal memory	Non-fasters	412.2 ± 165.6	566.6 ± 106.7	+154.4 ± 172.1	[+68.40, +240.40]	0.81	0.419	0.14
	Fasters	475.3 ± 229.1	564.2 ± 163.2	+88.9 ± 253.6	[−41.49, +219.29]			
Memory score (composite)	Non-fasters	430.3 ± 116.0	500.6 ± 80.8 ^a^	+70.2 ± 135.1	[+2.69, +137.71]	0.28	0.779	0.05
	Fasters	473.6 ± 137.8	524.4 ± 101.5 ^a^	+50.7 ± 153.8	[−28.38, +129.78]			

Notes. Values are means ± SD. CogniFit scores range 0–800 (higher = better cognitive performance). *n*: non-fasters = 18, fasters = 17. Parametric parameters (*): two-way mixed ANOVA; effect size = partial η^2^ (small: 0.01, medium: 0.06, large: 0.14). Non-parametric parameters: Mann–Whitney U on Δ scores; effect size r = rank-biserial correlation; STM = short-term memory. ^a^ Significantly different from Session 1 within group (*p* < 0.05, Wilcoxon signed-rank test). ^b^ Session 2 score significantly different from non-fasters (*p* < 0.05, Mann–Whitney U). The Session 2 between-group difference in phonological STM (^b^) reflects a numerical baseline differential that did not reach significance at Session 1 (*p* > 0.05) and is not attributable to the fasting intervention (Group × Time interaction: *p* = 0.982, r = 0.00).

**Table 3:** Descriptive statistics and Group × Time interaction results for CogniFit attention subdomains (scores 0–800).

Parameter	Group	Session 1 Mean ± SD	Session 2 Mean ± SD	Δ (Post − Pre) Mean ± SD	95% CI for Δ	Test Statistic (Z or F)	Exact *p*	Effect Size (r or η^2^*p*)
Divided attention †	Non-fasters	92.3 ± 245.7	81.4 ± 203.3 ^a^	−10.9 ± 202.5	[−112.14, +90.26]	0.17	0.869	0.03
	Fasters	8.0 ± 0.0	50.1 ± 139.9 ^a^	+42.1 ± 139.9	[−29.89, +114.01]			
Inhibition	Non-fasters	226.7 ± 114.0	245.9 ± 99.0 ^a^	+19.2 ± 108.7	[−35.12, +73.52]	0.2	0.843	0.03
	Fasters	178.1 ± 41.4	217.8 ± 95.3 ^a^	+39.6 ± 118.3	[−21.23, +100.43]			
Updating	Non-fasters	192.8 ± 137.8	246.2 ± 114.9 ^a^	+53.4 ± 114.8	[−3.96, +110.76]	1.35	0.176	0.23
	Fasters	158.8 ± 76.4	205.6 ± 120.1 ^a^	+46.8 ± 149.0	[−29.81, +123.41]			
Focused attention	Non-fasters	314.3 ± 148.1	385.2 ± 127.9 ^a^	+70.9 ± 177.9	[−17.99, +159.79]	1.27	0.204	0.21
	Fasters	311.3 ± 159.9	281.9 ± 122.8	−29.4 ± 202.7 ^b^	[−133.62, +74.82]			
Attention score (composite)	Non-fasters	206.6 ± 135.1	245.3 ± 97.8 ^a^	+38.8 ± 118.2	[−20.26, +97.86]	0.96	0.338	0.16
	Fasters	163.9 ± 62.5	188.8 ± 102.8 ^a^	+24.8 ± 134.5 ^b^	[−44.36, +93.96]			

Notes. Values are means ± SD. CogniFit scores range 0–800 (higher = better). *n*: non-fasters = 18, fasters = 17. All analyses: Mann–Whitney U test on Δ scores; effect size r = rank-biserial correlation (small: 0.10, medium: 0.30, large: 0.50). ^a^ Significantly different from Session 1 within group (*p* < 0.05, Wilcoxon signed-rank test). ^b^ Session 2 score significantly lower than non-fasters at the same time point (*p* < 0.05, Mann–Whitney U). † Divided attention: fasters baseline SD = 0 (all *n* = 17 scored 8/800).

**Table 4:** Descriptive statistics and Group × Time interaction results for Psychomotor Vigilance Test (PVT) parameters.

Parameter	Group	Session 1 Mean ± SD	Session 2 Mean ± SD	Δ (Post − Pre) Mean ± SD	95% CI for Δ	Test Statistic (Z or F)	Exact *p*	Effect Size (r or η^2^*p*)
Test duration (s) *	Non-fasters	122.80 ± 1.70	122.50 ± 2.00	−0.30 ± 2.70	[−1.65, +1.05]	0.18	0.673	0.01
	Fasters	122.40 ± 2.00	122.40 ± 1.70	+0.10 ± 2.70	[−1.29, +1.49]			
False starts (*n*)	Non-fasters	0.50 ± 1.47	1.56 ± 2.15 ^a^	+1.06 ± 2.10	[+0.01, +2.11]	0.1	0.921	0.02
	Fasters	0.88 ± 2.89	2.29 ± 3.64 ^a^	+1.41 ± 4.74	[−1.03, +3.85]			
Mean RT (ms)	Non-fasters	347.50 ± 79.60	319.10 ± 63.40 ^a^	−28.40 ± 44.90	[−50.84, −5.96]	0.31	0.754	0.05
	Fasters	329.80 ± 70.00	304.80 ± 46.40	−25.10 ± 94.00	[−73.43, +23.23]			
Attempts (*n*)	Non-fasters	22.22 ± 3.57	24.11 ± 2.27 ^a^	+1.89 ± 2.87	[+0.46, +3.32]	1.3	0.192	0.22
	Fasters	23.18 ± 3.40	23.06 ± 3.61	−0.12 ± 4.69	[−2.53, +2.29]			

Notes. Values are means ± SD; *n*: non-fasters = 18, fasters = 17. Parametric parameter (*): two-way mixed ANOVA; effect size = partial η^2^ (small: 0.01, medium: 0.06, large: 0.14); Non-parametric parameters: Mann–Whitney U test on Δ scores; effect size r = rank-biserial correlation; False starts = responses with latency < 100 ms, excluded from mean RT calculation; RT = reaction time. ^a^ Significantly different from Session 1 within group (*p* < 0.05, Wilcoxon signed-rank test).

## Data Availability

The data supporting the findings of this study are available upon reasonable request from the corresponding authors due to ethical and privacy restrictions related to participant confidentiality.
